# Predictors of real-life functioning in subjects with schizophrenia: A 4-year follow-up study

**DOI:** 10.1192/j.eurpsy.2021.135

**Published:** 2021-08-13

**Authors:** S. Galderisi, A. Mucci, D. Gibertoni, A. Rossi, P. Rocca, A. Bertolino, M. Maj

**Affiliations:** 1 Department Of Psychiatry, University of Campania “Luigi Vanvitelli”, NAPOLI, Italy; 2 Department Of Psychiatry, Univeristy of Campania Luigi Vanvitelli, Naples, Italy; 3 Department Of Biomedical And Neuromotor Sciences, University of Bologna, Bologna, Italy; 4 Department Of Biotechnological And Applied Clinical Sciences, Section Of Psychiatry, University of L’Aquila, L’Aquila, Italy; 5 Department Of Neuroscience, University of Turin, Torino, Italy; 6 Department Of Neurological And Psychiatric Sciences, University of Bari, Bari, Italy; 7 Department Of Psychiatry, University of Campania L. Vanvitelli, Naples, Naples, Italy

**Keywords:** psychopathology, neurocognition, social cognition, Functional Outcome

## Abstract

In a cross-sectional study, the Italian Network for Research on Psychoses (INReP) found that variables relevant to the disease, personal resources and social context explain 53.8% of real-life functioning variance in a large sample of community dwelling people with schizophrenia. In a longitudinal study, the INReP aimed to identify baseline predictors of main domains of real-life functioning, i.e. work skills, interpersonal relationships and everyday life skills, at 4-year follow-up. We assessed psychopathology, social and non-social cognition, functional capacity, personal resources, and context-related factors, as well as real-life functioning as the main outcome. We used structural equation modeling (SEM) and latent change score (LCS) model to identify predictors of real-life functioning domains at follow-up and changes from baseline in the same domains. Six-hundred-eighteen subjects took part in the study. Neurocognition predicted everyday life and work skills; avolition predicted interpersonal relationships; positive symptoms work skills, and social cognition work skills and interpersonal functioning. Higher neurocognitive abilities predicted the improvement of everyday life and work skills, as well as of social cognition and functional capacity; better baseline social cognition predicted the improvement of work skills and interpersonal functioning, and better baseline everyday life skills predicted the improvement of work skills. Several variables which predict important aspects of functional outcome of people with schizophrenia are not routinely assessed and are not systematically targeted by intervention programs in community mental health services. A larger dissemination of practices such as cognitive training and personalized psychosocial interventions should be promoted in mental health care.

**Disclosure:**

No significant relationships.

